# Potential of Gas-Propelled Aerosol Containing Synergized Pyrethrins for Localized Treatment of *Cryptotermes brevis* (Kalotermitidae: Blattodea)

**DOI:** 10.3390/insects14060522

**Published:** 2023-06-04

**Authors:** Babar Hassan, Chris Fitzgerald

**Affiliations:** Department of Agriculture and Fisheries, 50 Evans Road, Salisbury, QLD 4107, Australia; chris.fitzgerald@daf.qld.gov.au

**Keywords:** drywood termites, pyrethrin, PY mist, topical bioassay, residual toxicity, spot treatment

## Abstract

**Simple Summary:**

West Indian drywood termite (WIDT) is an invasive insect pest of wood and wood products in Australia, where confirmed infestations have been eliminated using mandatory structural fumigation; however, fumigation is costly, is disruptive, and does not prevent re-infestation. Localized treatments with registered insecticides are the most prevalent and cost-effective ways to treat drywood termite infestations; however, the use of liquid or foam insecticides does not ensure that an infestation has been completely eradicated. The amount of termite frass packed inside a gallery may prevent the chemical from reaching the target spot, and the treatment might not reach all termite galleries, especially during vertical application, which could be a barrier to complete eradication. The current study assessed the potential of aerosol-containing synergized pyrethrins as a spot treatment option for this pest. The results showed that wood surfaces treated with pyrethrins containing aerosol caused a high mortality of WIDT pseudergates in continuous and short exposures. Moreover, in different tests, an aerosol was passed through the loose fecal pellets packed in wooden or silicon tubing, causing high WIDT mortality.

**Abstract:**

The possibility of synergized pyrethrin-containing aerosol as a choice for spot-treating *C. brevis* in Australia was investigated in laboratory tests. Topical toxicity tests, where *C. brevis* pseudergates were subjected to multiple doses of pyrethrin mist insecticide, showed the concentration-dependent death of termites with a median lethal dose (LD_50_) of 193.16 µg. Residual toxicity tests, where the termites were exposed to wood surfaces treated with pyrethrin-containing aerosol, showed a rapid mortality in short and continuous exposures. Less than 20% of the termites survived even when the termites were exposed to a treated wood surface for a minute. All the termites died within 1–5 h in continuous exposure tests, depending on the age of the treated surface. In repellency tests, the termites tended to visit treated surfaces, causing an overall lower survival of the termites. The synergized pyrethrin-containing aerosol remained insufficiently volatile to produce the complete mortality of the termites even after 196 h when there was no contact with a treated surface. The number of termites that survived following the application of the synergized aerosol through a simulated wood gallery or silicon tubing with fecal pellets was also negligible, demonstrating the ability of the aerosol to penetrate through pellets and ultimately resulting in a distribution that is ideal for treatment in the termite galleries.

## 1. Introduction

*Cryptotermes brevis* (Walker, 1853), the West Indian drywood termite (WIDT), is a significant pest of timber in service inside buildings in many countries worldwide [1]. Drywood termite infestations are eliminated by treating a specific area of timber where termites are present in a structure called a localized treatment or using a whole-of-house treatment, where termites concealed in wood are killed by fumigating an entire building with, e.g., sulfuryl fluoride or by heating it to a specific temperature. Treating the whole structure is advantageous over a localized treatment as there can be several small colonies in a structure that are difficult to detect or access; however, this process is expensive and disruptive. Eliminating drywood termites with excess heat or cold also requires a considerable amount of energy, training, and investment in equipment. In localized treatments, conversely, an insecticide is injected into the termite galleries [2,3,4]. 

In Queensland, Australia, up until January 2021, *C. brevis* was managed by whole-of-structure fumigation at the Queensland Government’s expense under the WIDT Prevention and Control Program [2]. Several research projects were commenced in late 2022 to better equip Queensland’s pest control sector to identify, detect, and manage this pest in the future. An alternative to whole-of-house fumigation can be localized treatments with registered termiticides for drywood termites [3]. These will provide a cheaper and less disruptive option for a homeowner; however, the success rate of localized treatments with registered insecticides is low. In a localized treatment, the pest technician will drill a pattern of injection holes at close intervals (30 cm) across each board suspected of being infested, to treat as many termite gallery systems as possible. But, in many cases, even this does not guarantee the complete eradication of the infestation [5,6]. One possible impediment to full eradication is the amount of termite frass packed within a gallery that is hindering the delivery of a chemical to the target site. Most of the products registered against *C. brevis* in Australia are liquids [3]. Insecticides cannot be distributed evenly due to gravity, and it may be challenging to deliver liquid formulations to the termite colony core, especially during vertical application. Sometimes the amount of liquid used causes an issue with the timber being excessively wet, and it is challenging to drive liquids past fecal pellet accumulations [7,8]. Foam insecticides can mitigate the issue of excessive wetting, but they might not be able to spread from one point of entry throughout the gallery. Fecal pellet accumulations are also likely to impede foam formulations as well. Moreover, the architecture of a *C. brevis* gallery system may impede the complete coverage of the gallery system where termites are active when using foam or liquid formulations. This could be due to back pressure while applying liquid insecticides because of dead-end galleries and the narrow interconnecting galleries that pellets or termites can block [8,9]. Overcoming these issues would take considerable time, require numerous drill holes in termite-infested wood, and insecticide applications spread out over several months.

Pyrethrin is the active ingredient of pyrethrum, namely, an extract from flowers, and the insecticidal properties of pyrethrum have been known since ancient times [10]. It is a potent biopesticide that is a natural mixture of several compounds and has a very high efficacy against a broad spectrum of insect pests [11]. Pyrethrin-containing mist or aerosol formulations are approved commercially in Australia against various insect pests, including mosquitoes, silverfish, flies, fleas, beetles, grain storage pests, and many other pests on domestic and commercial premises [12,13]. Pyrethrin as a liquid, however, or pyrethrin-containing aerosol formulations, have not been approved against termites. This might be because it degrades quickly in light and does not accumulate or persist in the environment [10]. The current study examined the potential of a pyrethrin-containing aerosol for the localized treatment of *C. brevis*. This is a gas-propelled aerosol and two identical registered products in Australia are available, namely, Pestigas from BOC International and SupaPy from Supagas. These products consist of 4 g/kg of pyrethrin, 20 g/kg of piperonyl butoxide (as the synergist), and 100 g/kg of light hydrocarbon dissolved in 876 g/kg of liquid solvent-propellant CO_2_. The solvent-propellant, high-pressure (5000 kPa) liquid CO_2_ [13], is a low-cost solvent that may allow the pesticide to be forced through termite galleries in the timber and should readily penetrate through frass pellets leading to an ideal application. The irritating properties of pyrethrin [10] may also assist with increased exposure as termites try to escape. Pyrethrin is an insect neurotoxin and piperonyl butoxide acts by preventing metabolic detoxification of the insecticide. These products are labeled for use in domestic premises, hospitals, bakeries and food processing plants, hotels and motels, industrial sites, and animal housing due to low mammalian toxicity [12]. The objective of the current study was to determine the insecticidal activity and potential efficacy of pyrethrin-containing aerosol for the localized treatment of existing infestations of *C. brevis* in Australia.

## 2. Materials and Methods

### 2.1. Termites

Termites were collected and maintained in the laboratory as described previously by Hassan et al. [3]. Briefly, *C. brevis*-infested wood was collected from a building in Maryborough, Queensland. The collected material was transported in sealed plastic boxes to a laboratory at the EcoSciences Precinct in Dutton Park, Queensland, where the wood was kept at 27 °C and 70% RH until testing started. The fecal pellet shape was observed under a microscope to confirm the termite species [14,15]. *Cryptotermes brevis* fecal pellets are generally larger and pointed/elongated from one end compared to frass of most of the native drywood termite species present in Australia. Moreover, soldiers discovered in the colony after a wood dissection confirmed the species as *C. brevis*. Termites, and were carefully tapped into petri plates (with a 9 mm in diameter) from the infested wood after dissection using a chisel and hammer. The termites from several colonies were maintained together for a week in glass petri plates with two to three layers of black filter paper. Before commencing the experiments, groups of fifty healthy termites were placed into petri plates containing 2 to 3 hoop pine veneers (1.5–3 mm thick) and conditioned at 27 °C and 70% RH. Only the 3rd or 4th instar pseudergates belonging to eight different colonies with no evidence of wing buds were used in the experiments.

### 2.2. Chemicals and Associated Materials

PY insecticide mist, a formulated mist insecticide manufactured by C. Rudduck Pty. Ltd., New South Wales, Australia, was kindly provided by Mara Beddoes (TermitesRus). The product contained the same active ingredients of a pyrethrin-containing aerosol with the exception of carbon dioxide (CO_2_); therefore, this was used to determine the topical toxicity of pyrethrins to termites. It contained pyrethrin (4 g/L), piperonyl butoxide, and hydrocarbon liquid. It is commercially registered against several household and urban pests except for termites. Acetone used to dilute the PY mist was purchased from Sydney Solvents Pty. Ltd., New South Wales, Australia, while a light hydrocarbon liquid (Diggers Shellite) was sourced from the Bunnings Group Limited, Brisbane, Australia. Synergized pyrethrin aerosol, SupaPy, was provided by Supagas Pty. Ltd., Beenleigh, Queensland, Australia in an industrial-sized 6 kg aerosol cylinder. It contained 4 g/kg of pyrethrin, 20 g/kg of piperonyl butoxide as a synergist and 100 g/kg of light hydrocarbon dissolved in 876 g/kg of liquid solvent-propellant CO_2_. This was a self-propelled container requiring no auxiliary power and was fitted with a spray gun and nozzle to dispense an aerosol fog. Efficient atomization of the insecticide with a spray generates a particle size of ~5 microns which can remain airborne for a long period [13]. A spray gun kit with a SupaPy Spear Nozzle (737514) was provided by Supagas Pty. Ltd., Australia. Silicon air tubing (Aqua One 10471) with a 3–4 mm diameter and connectors (Aqua One 10468) were sourced from the Tech Den—Aquarium Store & Fish Experts, located in Caboolture, Queensland, Australia. Clear plastic containers were purchased from a Woolworths supermarket. 

In the further text, SupaPy is referred to as pyrethrin-containing aerosol or aerosol.

### 2.3. Topical Bioassay Using PY Mist

The PY mist was diluted using acetone as a solvent to prepare the desired concentrations. Based on preliminary experiments, the tested doses of pyrethrin against the drywood termites were 600, 400, 200, 80, 40, 20, and 13 µg per termite. Acetone and a weak solvent, namely, a light hydrocarbon liquid, were used as the control treatments. 

Pseudergates of *C. brevis* were topically treated with the doses mentioned above using a 1–10 µL micropipette to assess the chemical’s insecticidal potential. The termites were carefully picked up with a soft brush, and a total of 2 µL of the solution was applied to the thorax of each termite. The droplet was allowed to dry for 30 s, and the termites were placed in a plastic petri dish lined with moist black filter paper. The petri dishes and termites were held in a conditioning chamber for 24 h. Ten termites were treated with each concentration of solution. Each treatment was replicated three times. The number of live termites was counted after a 24 h post-application of the insecticide. Termites were considered dead when there was no movement upon touching with a soft brush or an inability to turn over after being touched.

### 2.4. Residual Exposure of C. brevis through Wood Surfaces Treated with Pyrethrin-Containing Aerosol

**Figure 1:**
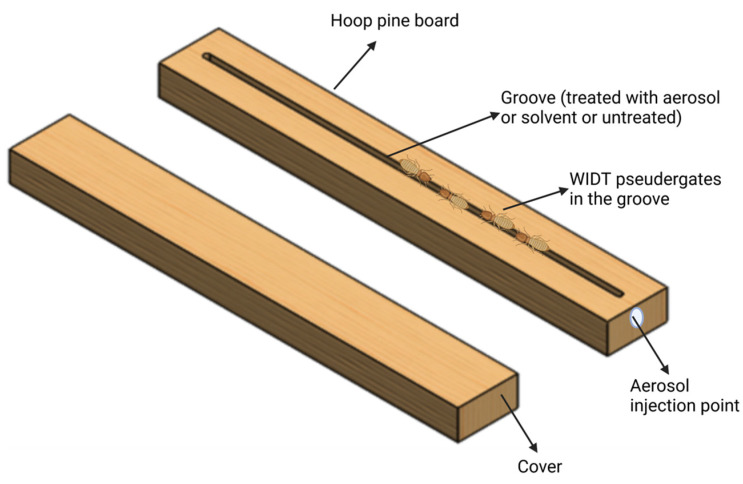
Wooden test unit for residual exposure of *C. brevis* through wood surface treated with synergized pyrethrins containing aerosol, solvent, or left untreated.

A simple bioassay was designed to expose the *C. brevis* in a gallery-within-wood scenario to evaluate the pyrethrin-containing aerosol treatment in the laboratory as described previously [3]. Briefly, using a band saw, 20 × 4 × 4 cm hoop pine boards were cut longitudinally in half. On the sawn surface of one board, a rounded groove of 4.5 mm × 4.5 mm × 18 cm was routed starting at the 1 cm point and finishing at the 19 cm point. A tiny hole was drilled at one end of the non-grooved board to administer an aerosol containing pyrethrin. (Figure 1). The aerosol was uniformly applied to the whole groove when required for testing. All units were stored in the conditioning chamber for a few weeks before testing to achieve 12–13% moisture content. All tests were performed at 27 °C, and 70% RH. 

*Cryptotermes brevis* pseudergates were exposed to the pyrethrin-containing aerosol for a short duration (1 and 5 min) or an extended duration (until all were dead), through contact with the treated surface. A G clamp was used to hold two wooden units (one on each end and one clamp in the center) to treat the grooves without termites. Following treatment, the units were placed in a conditioning room. For the continuous exposure tests, the treated surfaces of the wooden units were 1, 21 and 64 days old; however, for the short exposure tests, only one-day-old-treated surfaces of the wooden units were used. The continuous exposure test involved dismantling the treated units and releasing 10 pseudergates into the grooves. The units were re-clamped and stored in the conditioning room at 27 °C and 70% RH. There were three treatments (i.e., the pyrethrin-containing aerosol, and two controls), and each treatment was replicated four times. For the control treatments, the termites were continuously confined in the testing units without any treatment or were treated with the light hydrocarbon liquid. The number of alive termites was recorded after 30, 60, and 180 min post-exposure. 

For the short exposure experiments, 10 pseudergates from each unit were exposed for 1 and 5 min to the treated surfaces as described in the previous section. Following short exposures, the termites were removed from the grooves and placed on untreated filter paper in petri plates in the conditioning room. For the short exposure tests, the survival of the termites was recorded before transferring them to petri plates (time 0) and then after 0.5, 6, and 20 h post-exposure. For the control treatments, the termites were continuously confined in the testing units without any treatment for 5 min, then transferred to petri plates. All tests were performed at 27 °C, and 70% RH. 

### 2.5. Repellency of Surface Treated with Pyrethrin-Containing Aerosol against C. brevis

**Figure 2:**
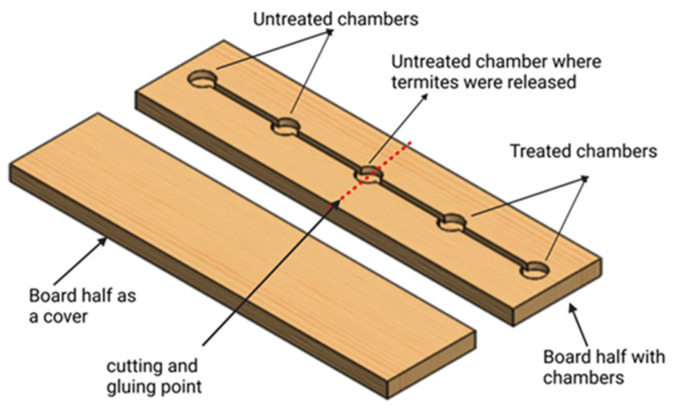
Example of wood test unit used in repellency test.

Repellency tests were conducted to determine if the *C. brevis* contacted or avoided the surfaces treated with the pyrethrin-containing aerosol. For this, we carved five compartments on a hoop pine board (2 × 4 × 20 cm), with each measuring 2 cm in diameter and 8 mm deep. The longitudinal connection between each chamber was made by a 4 mm simulated gallery level with the chamber floor. Two chambers on the far left were treated with the aerosol and placed in the conditioning chamber. To ensure the treatment of only two chambers, the board half containing the chambers was cut from the middle (chamber #3) vertically before the treatment and was rejoined using PVA glue after an application of the aerosol (where the preliminary experiments showed no effect of the PVA glue on termite survival). Thus, the central chamber contained the live termites, while the two chambers on the far right represented the untreated surface. The two chambers on the far left represented the treated surface (Figure 2). Ten *C. brevis* pseudergates were released into the middle chamber, and the top half of the board was clamped.

Termite locations and survival were recorded after 1, 2, 6, and 20 h by carefully dismantling the unit. The termites present in the middle chamber were excluded from the data analysis. For the control treatments, pyrethrin-containing aerosol was not applied to the wood surface, but the rest of the process was the same. Ten wooden units were utilized to repeat each treatment five times. We observed no evidence of cannibalism in the repellency experiments within 24 h of exposure; therefore, the repellency formula provided by Rust and Reierson [16] was slightly modified to calculate the repellency percentages [17]:

$$\mathrm{Repellency}\;\left(\%\right)=\frac{\;\left(\mathrm{Nc}-\mathrm{Nt}\right)}{\left(\mathrm{Nc}+\mathrm{Nt}\right)}\times 100$$

where Nc and Nt are the numbers of termites present in the untreated and treated chambers, respectively.

In a separate experiment, filter paper was used to assess the pyrethrin-containing aerosol’s ability to repel termites, as described by [3,17]. For this purpose, black filter paper discs (9 cm diameter) were cut in half. Aerosol was applied to one half but not to the other. After drying under a fume hood for 12 h, the halves were bonded together using adhesive tape affixed to the bottom side of the filter paper. They were then placed in a petri plate, and ten termite workers were introduced into each plate. The number of termites present on each filter paper and the termite survival after 1, 6, and 20 h were recorded. The repellency was calculated using the formula described above.

### 2.6. Vapor Toxicity of Synergized Pyrethrin-Containing Aerosol

Vapors of toxic pyrethrin-containing aerosol in a closed gallery system of *C. brevis* may cause termite mortality when the termites do not encounter the treated wood surface or chemical. To determine the vapor toxicity of the aerosol, *C. brevis* pseudergates were held under an aerosol-treated surface, and the surviving termites were counted at different time intervals. Petri plates containing two layers of untreated black filter paper were used to hold the termites and wood above the termites using a glass cylinder (with a diameter: 50 mm, and height: 30 mm). Hoop pine wood disks (60 mm diameter) were treated with synergized pyrethrin-containing aerosol and were used in the experiment when their surface was dry (after 1 h of treatment). Ten *C. brevis* pseudergates were placed on the filter paper inside the glass cylinder and the treated pine wood was placed on top of the cylinder where the treated surface of the disk faced downward toward the termites. The hoop pine wooden disks were left untreated for the control treatment, but the remainder of the steps were the same for the treated disks. A total of 30 termites were used in each treatment that was replicated three times. The survival of the pseudergates was recorded after 1, 17, 24, 40, 48, and 196 h.

### 2.7. Penetration of Pyrethrin-Containing Aerosol through C. brevis Fecal Pellets 

**Figure 3:**
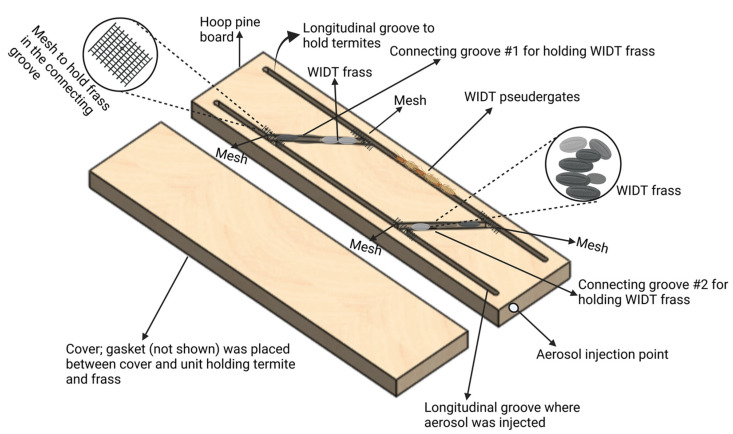
Example of the experimental setup used to determine aerosol penetration through fecal pellets within the wood scenario.

*Cryptotermes brevis* and other drywood termite frass can block the galleries hindering the effectiveness of spot treatments. The gallery system of *C. brevis* is composed of enormous chambers connected by narrow passageways. Loosely packed fecal pellets or termites can easily block these tiny passageways; therefore, the architecture of the drywood termite gallery system creates a blockage that may prevent the complete distribution of insecticides used for spot treatments [8,9]. Two separate experiments were carried out to ascertain the pyrethrin-containing aerosol penetration through *C. brevis* fecal pellets. In the first experiment, we evaluated the aerosol penetration in the laboratory while simulating dry wood termite damage in wood. A small wooden unit was designed to expose the termites, inject the aerosol, and determine the aerosol penetration through fecal pellets in a gallery-within-wood scenario. Hoop pine boards (150 × 40 × 200 mm) were cut longitudinally in half with a band saw. On the sawn surface of one board, two rounded grooves (4.5 × 4.5 × 180 mm) near the outer edges (5 mm from the edges) were routed in a longitudinal direction starting at the 10 mm point and finishing at the 190 mm point. One longitudinal groove was used to hold the termites during the experiment; the second was to inject the aerosol. Two vertical, rounded, connecting grooves (4.5 mm × 4.5 mm × 70 mm) designed to connect the longitudinal grooves were also created, which were used to hold *C. brevis* fecal pellets. A small hole was drilled at the starting point of one longitudinal groove as an aerosol injection port (Figure 3). Then, plastic mesh was glued at both openings of each connecting groove to keep the fecal pellets in only the connecting grooves and to prevent them from spreading in either of the longitudinal grooves. Half of the length of both connecting grooves was filled with an equal quantity (20 mg) of *C. brevis* fecal pellets. Ten *C. brevis* pseudergates were placed in one longitudinal groove. Both halves of the wood units were clamped together using G clamps. A gasket made up of butyl rubber was used as a mechanical seal to the space between two mating surfaces of the unit and to prevent the fecal pellets from falling out of the connecting gallery with pressure upon the aerosol application. The gasket also helped to limit aerosol penetration by only the connecting grooves, and not from the other surface of the unit. After clamping both halves of the wood unit, the pyrethrin-containing aerosol was injected using an injection nozzle in one longitudinal gallery without termites. The units were disassembled after application, and the number of live termites in the second longitudinal gallery where aerosol was not injected was counted after 5 and 10 min of application. The presence of pyrethrin-containing aerosol in the gallery with termites and its penetration through the fecal pellets was visually assessed.

**Figure 4:**
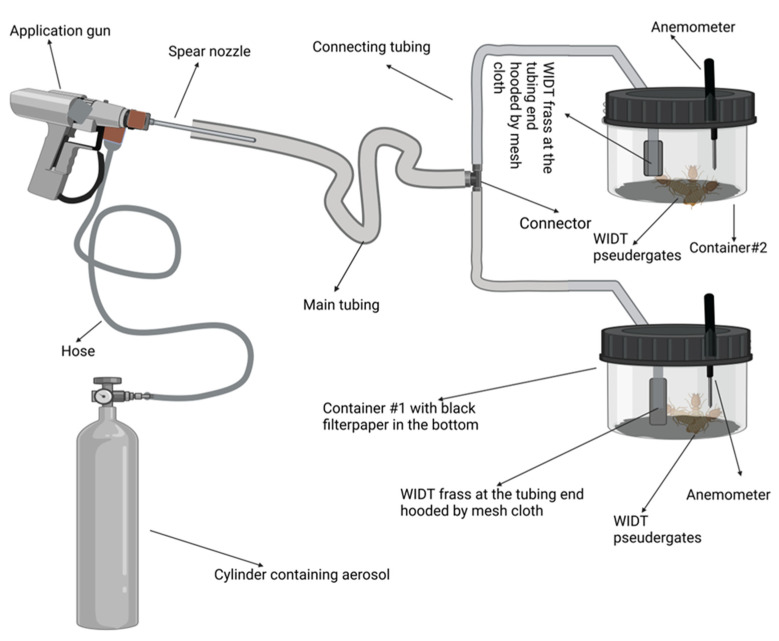
Example of the experimental setup used to determine aerosol penetration through fecal pellets in the silicon air tubing.

In the second experiment, penetration of the pyrethrin-containing aerosol through fecal pellets was assessed using silicon airline tubing (Aqua One 10471). Two round clear 500 mL plastic containers with a double layer of black filter paper were used to expose *C. brevis* pseudergates to the aerosol and its penetration through the termite fecal pellets. For this purpose, two holes were drilled in the lid of each plastic container to attach connecting tubing (60 cm long) and an anemometer to each container. A hotwire thermal anemometer (Testo 450i) with a measuring range of 0–30 m/s was used to measure the air speed in the container upon an injection of aerosol. Mesh cloth was glued to one end of each connecting tube used to hold the termite fecal pellets. Both connecting tubes were filled with fecal pellets up to 10 cm from the end. The other end of each connecting tube was connected to the main tubing using a three-way connector. Ten termites were placed in each container, and pyrethrin-containing aerosol was injected from the main tubing (Figure 4). The airspeed was recorded to confirm the aerosol penetration, and the termites’ survival was recorded post-application. The experiment was replicated three times, and there was no aerosol application in the control treatment.

### 2.8. Data Analysis

The datasets generated were first tested for normality and homogeneity of variance using the Kolmogorov–Smirnov D test. The experimental mortality from PY mist bioassays was corrected with the Abbott’s formula before the calculation of the lethal doses LD_50_ and LD_90_ with an associated 95% confidence interval (CL). Chi-squares were estimated using a Probit analysis and then the mortality data were analyzed using a one-way analysis of variance (ANOVA) followed by a Tukey’s HSD test. Data from the aerosol toxicity and repellency studies were subjected to a repeated measure analysis of variance. Each analysis of variance was followed by a Tukey’s HSD test for significant differences between the means. The level of significance was set at α = 0.05. All the statistical analyses were carried out using GraphPad Prism Version 7, Minitab 16, and Jamovi 16.9 under the AGPL3 license. 

## 3. Results

### 3.1. Topical Bioassay

**Figure 5:**
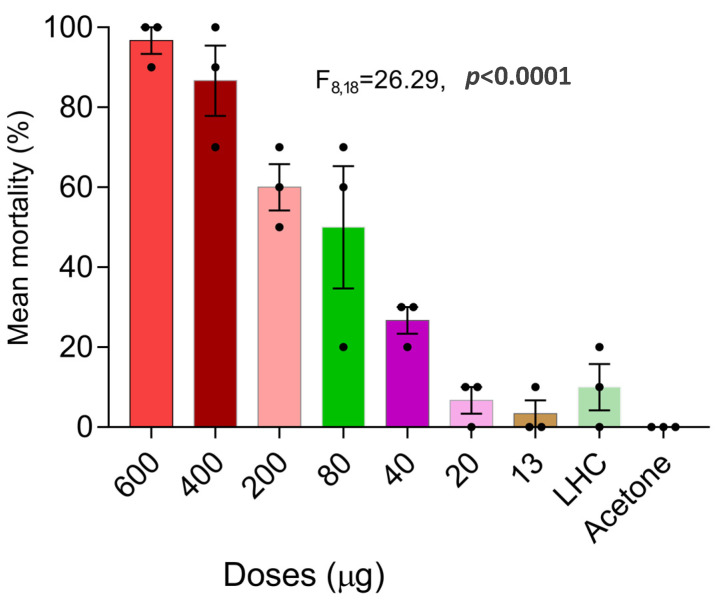
Mean mortality of *C. brevis* pseudergates after 24 h exposed to different doses of pyrethrin and control treatments in topical bioassay (LHC: light hydrocarbon). Error bars represent the standard error by Tukey’s HSD Test at α = 0.05.

The bioassay results using the acetone-diluted PY insecticide mist are shown in Table 1 and Figure 5. Pseudergates of *C. brevis* subjected to multiple doses of pyrethrins showed a concentration-dependent mortality. When the pseudergates were treated with the maximal dose of 600 µg pyrethrin, the highest mortality rate (97%) was observed, and the median lethal dose (LD_50_) observed was 193.16 µg. A one-way ANOVA revealed a significant difference in the efficacy of the treatments against the drywood termites. When termites were treated with acetone alone, there was no mortality observed; however, after treatment with a light hydrocarbon (Shellite), 10% of the drywood termite pseudergates died.

### 3.2. Short and Continuous Residual Exposures 

**Figure 6:**
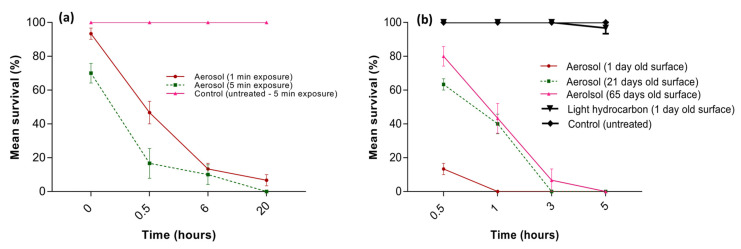
Mean survival (%) of *C. brevis* pseudergates in short (**a**) and continuous (**b**) exposure tests. In the short exposure test, termites were housed for 1 and 5 min on the one-day-old aerosol-treated surfaces in the wooden test units before being transferred to petri plates containing filter papers. In the continuous exposure test, the termites were kept until all died on 1-, 21-, and 65-day-old aerosol-treated surfaces in the wooden test units. Error bars represent standard error by Tukey’s HSD test at α = 0.05.

Exposure of termites to pyrethrin-containing aerosol through wood-treated surfaces resulted in significantly lower termite survival than the control treatments in one- and five-minute short exposure tests (Figure 6a). The termite survival was significantly lower in the first 30 min when these were exposed to the treated surface for five minutes than the survival of those termites which were exposed to the treated surface for one minute; however, the survival of termites at one and five minutes of exposure did not differ significantly at six and twenty hours following exposure. In the short exposure tests, overall, the effect of treatment (F_1,8_ = 139.8; *p* < 0.001), exposure time (F_1,8_ = 19.0; *p* = 0.002), and treatments × time (F_1,8_ = 19.0; *p* = 0.002) were significant. All termites exposed to the untreated or light hydrocarbon-treated wood surfaces survived.

In continuous exposure tests, a one-day-old pyrethrin-containing aerosol-treated surface provided a faster kill of termites compared to the 21- and 65-days-old-treated surfaces. Overall, the effect of treatment (F_2,18_ = 298.7; *p* < 0.001), age of surface (F_2,18_ = 47.1; *p* < 0.001), and treatments × surface age (F_4,18_ = 834.3; *p* < 0.001) were all significant. All termites exposed to the untreated or LHC-treated wood surfaces survived. All termites were dead within one hour of exposure when the termites were confined on one-day-old-treated surfaces; however, more than 40% of the termites survived after one hour of exposure when exposed to 21- and 65-days-old-treated surfaces. On the 21- and 65-days-old-treated surfaces, no termites survived after 3 and 5 h of exposure, respectively. Moreover, the survival of the termites when exposed to 21- and 65-day-old surfaces were not significantly different (t_2_ = 1.18; *p* = 0.17). 

### 3.3. Repellency Assay

**Figure 7:**
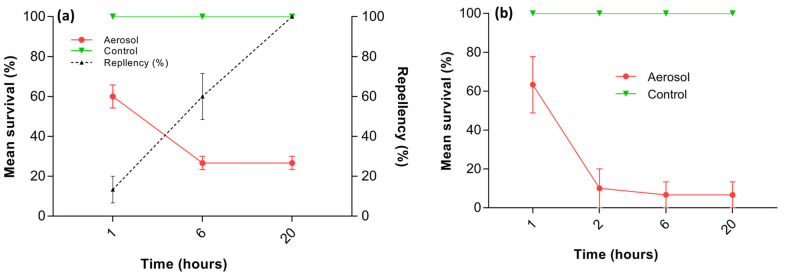
Mean termite survival and repellency (%) of pyrethrin-containing aerosol-treated wood surfaces to *C. brevis* pseudergates after 1, 6, and 20 h of exposure in filter paper test (**a**) and mean survival (%) of *C. brevis* pseudergates in five chamber tests at different time intervals (**b**). Error bars represent standard error by Tukey’s HSD test at α = 0.05.

The surface treatment of a filter paper with an aerosol-containing pyrethrin significantly impacted the termite survival compared to the control treatments (F_2,3_ = 90.5; *p* = 0.002). Sixty percent of the termites survived after being exposed for an hour, indicating that some of the termites visited the treated half of the filter paper; however, after six hours of exposure, the survival of the termites decreased further, and the repellency rate was around 60%. After this, the repellency rate reached 100%, and all termites that had survived tended to remain on the untreated half of the filter paper (Figure 7a). Similarly, the surface treatment of wood with the pyrethrin-containing aerosol also significantly impacted the termite survival compared to the control treatment (F_1,4_ = 465.0; *p* < 0.001). After an hour of exposure, the mean survival rate of termites exposed to the gallery’s treated and untreated surfaces was 60%. Termites visited the treated surface of the wood in the first hour; however, the termite survival was less than 15% after 2, 6, and 20 h of exposure, but similar at each time. This suggests the termites tended to remain on the untreated surface of the gallery in the wood after initial visits (Figure 7b).

### 3.4. Vapor Toxicity Assay

**Figure 8:**
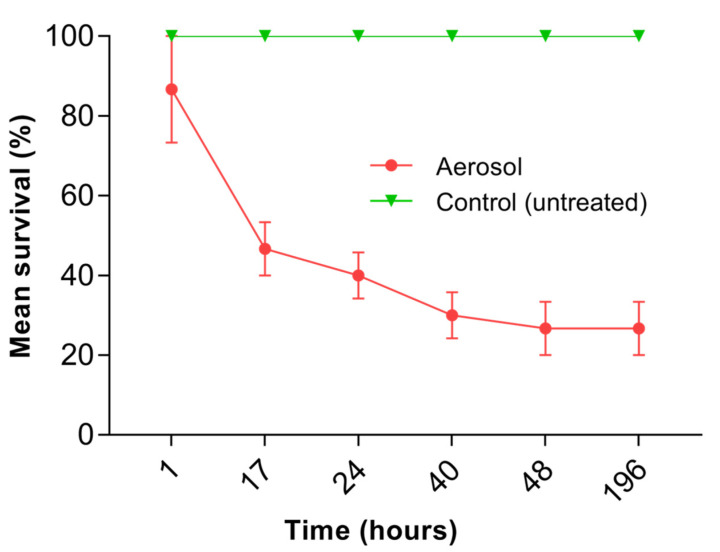
Mean survival of termites exposed to the airspace below the one-day-old pyrethrin-containing aerosol deposits on the wood. Error bars represent standard error by Tukey’s HSD test at α = 0.05.

When termites were exposed to the airspace beneath the aerosol-treated surface, a significantly lower survival was seen compared to the control treatment (F_1,4_ = 79.8; *p* < 0.001); however, the pyrethrin-containing aerosol did not have enough volatility to produce a 100% kill of termites even after 196 h. Survival of the termites decreased to 40% after 24 h of exposure, but the termite survival was significantly similar after 24 h of exposure to 196 h (Figure 8).

### 3.5. Penetration of Pyrethrin-Containing Aerosol through C. brevis Fecal Pellets

**Figure 9:**
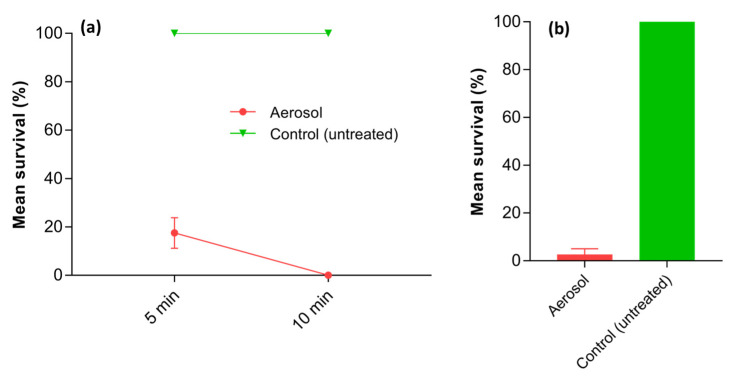
Mean termite survival after pyrethrin-containing aerosol exposure following its forced entry by fecal pellets in wood galleries (**a**) and silicon air tubing (**b**). Error bars represent standard error by Tukey’s HSD test at α = 0.05.

The complete mortality of termites in the gallery within the wood after the pyrethrin-containing aerosol application shows that it successfully penetrated through the fecal pellets positioned in the connecting galleries in the wooden unit to an opposite longitudinal gallery containing termites. The presence of aerosol on the opposite longitudinal gallery was also physically confirmed upon disassembly post-application. All the termites were dead within 10 min of the application of pyrethrin-containing aerosol, and all the termites survived in the control treatment (F_1,6_ = 841; *p* < 0.001) (Figure 9a).

The number of termites that survived following the application of the pyrethrin-containing aerosol through silicon tubing with fecal pellets at both ends was also negligible (i.e., 3%; only one termite survived out of 30), demonstrating the ability of the aerosol to penetrate through loose pellets, and this may result in a distribution that is ideal in the termite galleries (Figure 9b). All the termites survived in the control treatment (F_1,6_ = 123.4; *p* < 0.001).

## 4. Discussion

In Australia, the development of pyrethrins-based commercial pest control aerosol products and the establishment of a pyrethrum industry have historical ties. The Commonwealth Industrial Gases (CIG) introduced this innovative CO_2_-propelled aerosol containing pyrethrins to the pest management market in 1976. Since then, it has been widely used to control a variety of insect pests due to its low mammalian toxicity, non-flammability, ultra-fine particle size, container size, automatic operation and very high pressure (~5000 kPa) [18]. Pyrethrins interfere with the voltage-gated sodium ion channel in insect nerve cells, causing recurrent, prolonged nerve firings that lead to hyperexcitation [19]. Toxicity of synergized pyrethrin has been reported against several insect pests in several studies [20,21,22]; however, only a few studies have reported toxicity and repellency of pyrethrins against termites [23,24,25,26]. This could be because pyrethrins are easily photodegraded under sunlight, resulting in short residual effects, and this has limited their use in outdoor applications [21,27]; however, spot applications for the eradication of *C. brevis* do not require stability in light, as this pest termite tends to stay inside wood and in houses [2]. Compared to our study, a higher dose (6.39 mg/L) was required to kill a 50%-exposed population of *Coptotermes formosanus* Shiraki, a subterranean termite, as was observed in a previous study [23]; however, pyrethrin in the previous study was tested without a synergist. Our results also showed that drywood termites were less susceptible to pyrethrins followed by dampwood compared to the subterranean termite (*Microcerotermes* spp.). Drywood and dampwood termites are more prominent in size and body weight than subterranean termites, which may be linked with pyrethrin susceptibility. The smaller surface-to-volume ratio of larger individuals could restrict insecticide distribution throughout the cuticle following a topical application and lead to less or slower cuticle penetration [28].

For the spot treatments of drywood, the termite residual activity of insecticides is crucial [8]. While applying an insecticide, termites in the gallery system may not come into direct contact with the insecticide; however, they may encounter residual insecticides in the galleries sometime after application. Insecticide residue may also limit re-infestations of drywood termites [8]. The results of the residual exposure tests demonstrated that pyrethrin-containing aerosol is a highly quick-acting natural insecticide and that it killed termites considerably faster than the imidacloprid and fipronil foam, which are approved insecticides for the use against drywood termites in Australia [3]. Previous studies have reported that it took two days for all termites to die when exposed to a fipronil foam-treated surface for 4 or 8 h, and that imidacloprid-treated wood surface took longer still [3]. Our results also suggest that pyrethrin-containing aerosol has some residual life on a wood surface which slightly decreases as the surface ages. Residues of the pyrethrin-containing aerosol were active even after 64 days of age of a treated surface. A previous study reported that a natural pyrethrin emulsion was in a very low quantity in the soil after being stored at 60 °C for 2 months [23]; however, under field conditions, in termite-infested houses, a higher temperature and exposure to sunlight is unlikely to happen. Moreover, light hydrocarbon may provide a protective film to protect pyrethrin from degradation. Consequently, this warrants further studies.

Pyrethrin is also a potent insect repellent when applied at low doses and its repellency of pyrethrins against insect pests has been recognized for many years [10]. However, the lower survival of *C. brevis* pseudergates in the repellency tests showed that the termites made some initial visits on the treated surface that were enough to cause mortality because of the quick action of the pyrethrins. We observed a very low survival of *C. brevis* in both repellency tests. Only a few studies have reported the repellency of pyrethrins, and their potential use for the prevention of drywood termite infestations. Scheffrahn et al. reported no or only a few nuptial chambers of *C. brevis* and live termites in two different studies on wood blocks having a surface treated with a silica gel-pyrethrin dust compared with neighboring controls [26,29]. Our results showed that the tested pyrethrin-containing aerosol penetrated through fecal pellets successfully compared to the foam insecticide. This shows that the aerosol may lead to a complete treatment of the termite’s gallery system that might not be possible through foam and liquid insecticides. This may also solve the problem of back pressure as observed with liquid insecticides due to its high pressure and leading to the treatment of the narrow interconnecting galleries that pellets or termites can block.

The current findings imply that the pyrethrin-containing aerosol can be injected into a termite gallery and should penetrate loose frass, resulting in an optimal distribution. Pyrethrin-containing aerosols have been used by the pest control industry in Australia for a considerable amount of time; most technicians have had some exposure, and most businesses have it as a part of their arsenal [12]. The insecticide could be applied quickly and with less equipment than liquid insecticides. Pyrethrin-containing aerosols, however, as with other spot treatments, will only be useful where live termites have been detected or are suspected [3]. In addition to visual inspections, locating drywood termite infestations utilizing microwave- or acoustic-based instruments may simplify the location of a termite gallery system when live termites are not clearly visible [30].

## 5. Conclusions

Our results showed that the pyrethrin-containing aerosol caused very rapid mortality in *C. brevis*. Very short exposures of drywood termites on aerosol deposits on the wood surface were needed to achieve a higher termite mortality in both short- and continuous-exposure tests; however, the toxicity of deposited residues slightly decreased with the age of the treated surface. Our results also showed that the aerosol could penetrate through *C. brevis* fecal pellets and may result in an ideal distribution in the termite galleries to eradicate target termites. However, field studies are needed to test aerosols as localized treatments and termite detection devices to discern termite gallery systems.

## Figures and Tables

**Table 1:** Lethal doses for *C. brevis* exposed to a PY insecticide mist at different doses in topical bioassay.

LD_50_ (95% CL) (µg)	LD_90_ (95% CL) (µg)	Slope ± SE	χ^2^ (df)	*p*-Value
193.16 (158.98–235.04)	408.67 (347.54–501.43)	1.14 ± 0.13	23.86 (6)	0.001

LD: lethal doses killing 50% (LD_50_) or 90% (LD_90_) of the exposed population; CL: confidence limit; SE: standard error; χ^2^: chi square; df: degrees of freedom.

## Data Availability

All relevant data are within the paper.
